# Predicting the Response of High Frequency Spinal Cord Stimulation in Patients with Failed Back Surgery Syndrome: A Retrospective Study with Machine Learning Techniques

**DOI:** 10.3390/jcm9124131

**Published:** 2020-12-21

**Authors:** Lisa Goudman, Jean-Pierre Van Buyten, Ann De Smedt, Iris Smet, Marieke Devos, Ali Jerjir, Maarten Moens

**Affiliations:** 1 Department of Neurosurgery, Universitair Ziekenhuis Brussel, Laarbeeklaan 101, 1090 Brussels, Belgium; 2 Center for Neurosciences (C4N), Vrije Universiteit Brussel, Laarbeeklaan 103, 1090 Brussels, Belgium; ann.desmedt@uzbrussel.be; 3 STIMULUS Consortium (reSearch and TeachIng neuroModULation Uz bruSsel), Universitair Ziekenhuis Brussel, Laarbeeklaan 101, 1090 Brussels, Belgium; 4 Pain in Motion International Research Group, Laarbeeklaan 103, 1090 Brussels, Belgium; 5 Multidisciplinary Pain Center, AZ Nikolaas, Moerlandstraat 1, 9100 Sint-Niklaas, Belgium; info@drvanbuyten.be (J.-P.V.B.); Iris.Smet@aznikolaas.be (I.S.); Marieke.DeVos@aznikolaas.be (M.D.); Ali.Jerjir@aznikolaas.be (A.J.); 6 Department of Physical Medicine and Rehabilitation, Universitair Ziekenhuis Brussel, Laarbeeklaan 101, 1090 Brussels, Belgium; 7 Department of Radiology, Universitair Ziekenhuis Brussel, Laarbeeklaan 101, 1090 Brussels, Belgium

**Keywords:** pain, prediction, 10 kHz spinal cord stimulation, responders, machine learning

## Abstract

Despite the proven clinical value of spinal cord stimulation (SCS) for patients with failed back surgery syndrome (FBSS), factors related to a successful SCS outcome are not yet clearly understood. This study aimed to predict responders for high frequency SCS at 10 kHz (HF-10). Data before implantation and the last available data was extracted for 119 FBSS patients treated with HF-10 SCS. Correlations, logistic regression, linear discriminant analysis, classification and regression trees, random forest, bagging, and boosting were applied. Based on feature selection, trial pain relief, predominant pain location, and the number of previous surgeries were relevant factors for predicting pain relief. To predict responders with 50% pain relief, 58.33% accuracy was obtained with boosting, random forest and bagging. For predicting responders with 30% pain relief, 70.83% accuracy was obtained using logistic regression, linear discriminant analysis, boosting, and classification trees. For predicting pain medication decrease, accuracies above 80% were obtained using logistic regression and linear discriminant analysis. Several machine learning techniques were able to predict responders to HF-10 SCS with an acceptable accuracy. However, none of the techniques revealed a high accuracy. The inconsistent results regarding predictive factors in literature, combined with acceptable accuracy of the currently obtained models, might suggest that routinely collected baseline parameters from clinical practice are not sufficient to consistently predict the SCS response with a high accuracy in the long-term.

## 1. Introduction

Spinal cord stimulation (SCS) is considered an effective treatment option for patients with chronic pain, among which patients with failed back surgery syndrome (FBSS) [1,2,3]. Recent studies clearly indicated that SCS is cost-effective when considering a long-term time horizon, particularly for the management of FBSS [4]. Nevertheless, one of the main challenges in neuromodulation is to predict which patient will benefit from SCS treatment before implantation takes place. Mekhail et al. (2020) revealed that age, sex, depression, presence of neuropathic pain, and presence of spine-related pain are important factors for predicting the analgesic success of SCS [5]. Other studies have denoted the importance of psychological factors [6,7] and sleep interference [8] in relation to the response of SCS, whereby mainly univariate/multivariate logistic regression models and correlation analyses were applied to select relevant predictors. Additionally, the wide variety of SCS stimulation paradigms currently used in clinical practice made the search towards consistent predictive factors to determine the long-term response of SCS even more complicated.

A possible way to tackle this problem is by applying several machine learning techniques besides correlation analyses and logistic regression models. The central idea behind machine learning, which is an application of artificial intelligence, is that models are constructed by a set of data points and trained through mathematical and statistical approaches that ultimately enable the prediction of previously unseen data without explicitly being programmed to do so [9]. In contrast to traditional approaches, these machine learning models learn from examples and are not programmed with predefined rules [10]. Nowadays, machine learning techniques are broadly implemented in medicine and more specifically to aid clinicians with the prognosis, diagnosis, and treatment of patients, but also to optimize the workflow of clinicians [10,11].

In this study, machine learning techniques were applied to gain further insight into the preoperative factors related to a successful outcome with SCS. The goal was to create accurate prediction models with relevant predictors that would enable clinicians to predict whether a patient will be a responder or non-responder before definitive SCS implantation. To limit heterogeneity due to the stimulation paradigms, the focus of this retrospective study is to predict responders for high frequency SCS at 10 kHz (HF-10) therapy by only using data before definitive SCS implantation. This type of high frequency sinusoid stimulation previously demonstrated a persistent positive effect on pain [12,13], whereby the putative mechanisms are considered to be segmental [14]. Therefore, the aim of this study was to predict responders to HF-10 SCS with data available before definitive SCS implantation in patients with FBSS.

## 2. Experimental Section

### 2.1. Participants

Data of patients who were suffering from FBSS and treated with HF-10 SCS were included in this retrospective study. Databases of the two participating hospitals (AZ Nikolaas and UZ Brussel) were reviewed for SCS trials that occurred between July 2016 and January 2020. FBSS was defined as the surgical end-stage after one or several operative interventions on the lumbar neuroaxis, indicated to relieve lower back pain, radicular pain, or the combination of both without a positive effect [15]. Inclusion criteria consisted of (1) FBSS with pain in the back and/or leg(s), (2) age above 18 years, and (3) treatment with HF-10 SCS. Patients who had a negative SCS trial were not included in this study. Data of all patients that fulfilled these inclusion-criteria between July 2016 and January 2020 was extracted, and no patients were omitted (no selection bias).

The study protocol of this retrospective study was approved by the ethics committee of both AZ Nikolaas and Universitair Ziekenhuis Brussel (B.U.N. 1432020000129). The study protocol was registered on clinicaltrials.gov (NCT04500691).

### 2.2. Data Collection

Data was extracted from the individual patient files by trained medical staff of both hospitals. Based on available literature [5,16,17] and our clinical experience, the following data was extracted: date of SCS trial, age at SCS implantation, sex, pain intensity score for low back pain at baseline and at the last available follow-up visit, pain intensity score for leg pain at baseline and at the last available follow-up visit, predominant low back or leg pain (predominant pain location), medication at baseline and at the last available follow-up visit, Oswestry Disability Index (ODI) score at baseline and at the last available follow-up visit, body mass index (BMI), mean arterial pressure, number of previous spine surgeries, pain onset and pain duration, smoking status, work situation at baseline, socioeconomic status, and date of the last follow-up visit.

### 2.3. Data Processing

To facilitate data handling, variables were coded in predetermined categories. Pain intensity was categorized according to the International Classification of Diseases 11th Revision (ICD-11) in mild, moderate, or severe pain [18]. Predominant pain location was coded into three categories namely ‘back’, ‘leg’, or ‘back and leg’. Pain onset was categorized as ‘remaining’, ‘recurrent’, or ‘new’. Work status was categorized as ‘working’, ‘working without a financial compensation’, ‘not working and receiving a worker’s compensation’, or ‘not working and not receiving a worker’s compensation’. Socioeconomic status was divided into ‘married’, ‘single’, ‘living together’, or ‘living apart together’. Additionally, a binary variable was created to indicate whether the patient has children or not. Medication use was converted into a single numerical output that represented the negative impact of each medication [19] with the aid of the Medication Quantification Scale III (MQS). The MQS is designed to quantify pain medication regimens in a wide variety of pain conditions [20]. For each medication, a MQS score is calculated by multiplying a detriment weight for a given pharmacologic class with a score for dosage [21].

The outcome variable of this study, i.e., a responder to HF-10 SCS, is defined as a patient who has 50% pain relief for the predominant pain location at the last follow-up visit compared to baseline. This was calculated as follows: ((baseline pain intensity − follow-up pain intensity)/baseline pain intensity) × 100. The 50% cut-off value is the well-established definition of success worldwide and used as reimbursement criteria. Additionally, the outcome variable was also defined as a patient who has 30% pain relief, which is in line with the minimal clinical important difference for pain intensity [22]. Finally, the outcome variable was also defined as a patient who has at least 41.2% reduction in pain medication use, which is in line with the minimal clinical important difference for medication use in this population [23].

### 2.4. Statistical Analysis

All analyses were performed in R Studio version 1.2.5019 (R version 3.6). Descriptive statistics are provided as mean (±standard deviation), median (interquartile range) or as number of observations (percentage). Unless stated otherwise, *p*-values of 0.05 or less were considered statistically significant. There was no imputation for missing data.

The complete dataset was randomly divided into a train set and validation set. Machine learning techniques were applied on an 80% train set and tested on a 20% validation set to determine relevant predictors for being a responder (both 50% and 30% pain relief) to HF-10 therapy. Prediction accuracy ((true positives + true negatives)/total number of positives and negatives) and area under the curve of the receiver operating characteristics (ROC) curves were used to compare the performance of the different models (caret and pROC packages).

During feature selection, both the Spearman rank correlations and univariate analysis were applied to determine the variables that were related to percentage pain relief between the last follow-up and baseline for the predominant pain location. To avoid missing some potentially interesting variables, variables that revealed a correlation or association with *p* < 0.10 rather than *p* < 0.05 were used in further analyses.

Given that the prediction of a responder is a binary classification problem, logistic regression was considered suitable with responder status as outcome variable and patient specific characteristics at baseline as explaining variables. Model building was restricted to main effects and two-way interaction terms. A combined forward and backward stepwise elimination procedure (based on Akaike information criterion (AIC) values) was used to select the most optimal model. If a higher order interaction term needed to be included in the model, the main effects remained in the model as well. Furthermore, linear discriminant analysis (MASS package), classification and regression trees (caret package), random forest (randomForest package, 2 variables available for splitting at each tree node (mtry = 2)), bagging (randomForest package, (mtry = number of predictors)), and boosting (gbm package) were applied to this dataset. For the classification trees, pruning was performed with the rpart method, which consists of testing different values for the complexity parameter to find the most optimal complexity parameter that maximizes the cross-validation accuracy and results in the best tree model to explain the data (10-fold cross validation, 10 possible complexity parameters). Boosting was performed with 10-fold cross-validations as the resampling scheme.

## 3. Results

### 3.1. Patient Characteristics

**Figure 1:**
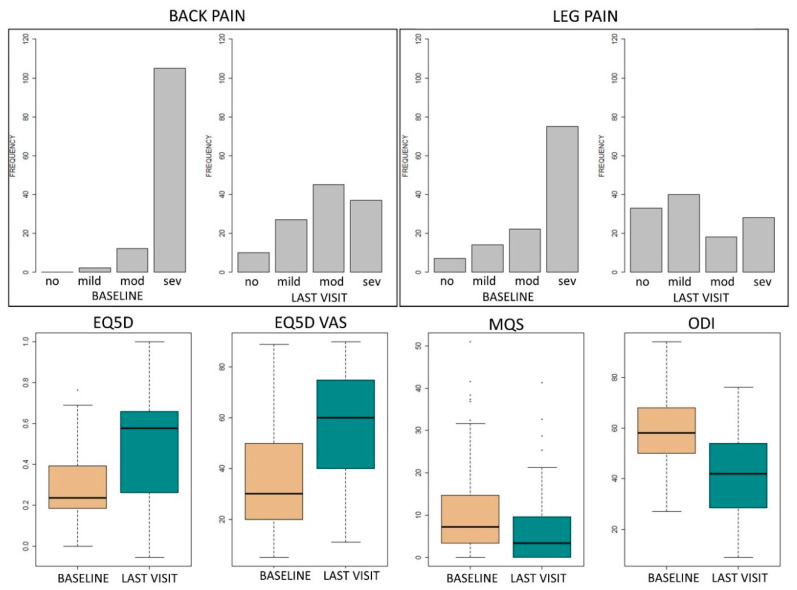
Barplots and boxplots of pain intensity categories, EQ5D, EQ5D VAS, MQS, and ODI scores at baseline and last visit. Abbreviations— EQ5D: the EuroQol with five dimensions and three levels; mod: moderate; MQS: medication quantification scale; ODI: Oswestry Disability Index; sev: severe; VAS: visual analogue scale.

In this retrospective study, data from 119 patients who underwent an SCS trial between July 2016 and January 2020 was used. The database was locked in June 2020. The median time between SCS trial and the last follow-up visit was 591.5 (Q1–Q3: 312.5–815.8) days. In total, 78 female patients (65.55%) and 41 male patients (34.45%) participated in this study with a mean age of 51.5 years (SD: 10.11 years). Additional patient characteristics can be found in Table 1. Figure 1 is presenting scores at baseline and at the last visit for all patients.

**Figure 2:**
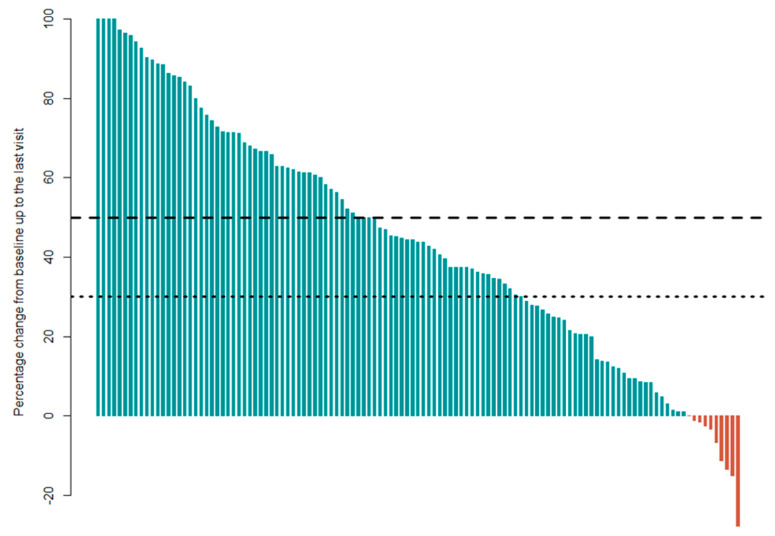
The waterfall plot presents the individual responder status of every patient (*n* = 119). A responder is a patient who demonstrated at least 50% pain relief (above the dashed line) or 30% pain relief (above the dotted line) for the predominant pain location at the last follow-up visit compared to baseline. If a bar is located below zero (red bars), then this patient has more pain at the last follow-up visit compared to baseline. Bars above zero (green bars) indicate less pain at the last follow-up visit compared to baseline.

The first definition of a responder was defined as a patient who has at least 50% pain relief for the predominant pain location at the last follow-up visit compared to the baseline. In total, 52 patients could be classified as a responder, while 67 patients were considered ‘non-responders’. Based on the definition of a 30% pain relief to be a responder, 79 patients could be classified as a responder while 40 patients were considered ‘non-responders’. Figure 2 presents a waterfall plot of the responder status of all participants. The third definition of a responder was defined as a patient who has at least 41.2% reduction in pain medication use at the last follow-up visit compared to baseline. With this definition, 60 patients could be allocated as ‘non-responders’ and 58 patients as responders.

Subsequently, the complete dataset was randomly divided into a train set (80% of the data, *n* = 95) and validation set (20% of the data, *n* = 24) for further analyses.

### 3.2. Feature Selection

**Figure 3:**
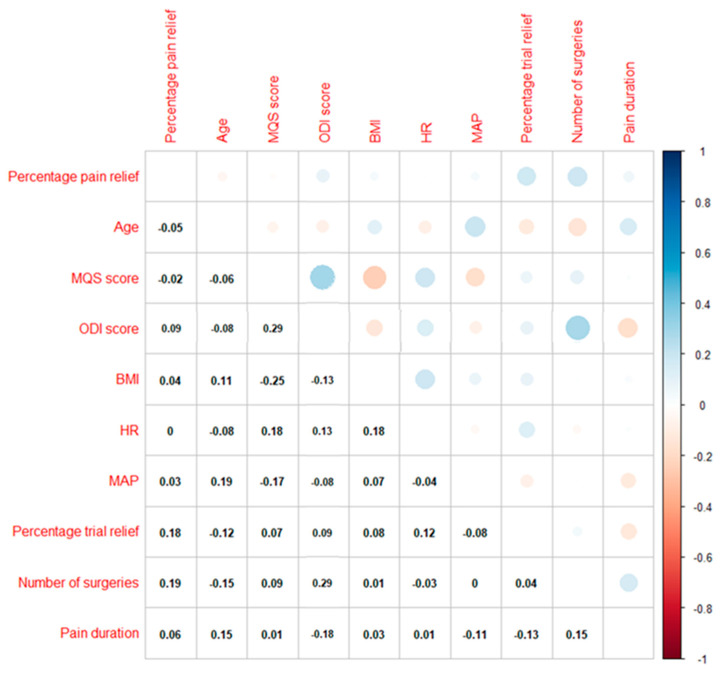
Correlation plot between percentage pain relief and explanatory variables for the training data set. Correlation coefficients are ranging from −1 (red) to +1 (blue). The strength of the correlation is represented in the size of the circles. Abbreviations. BMI: body mass index, HR: heart rate, MAP: mean arterial pressure, MQS: Medication Quantification Scale III, ODI: Oswestry Disability Index.

When evaluating variables related to responders for pain relief, Spearman rank correlations only revealed two statistically significant correlations namely between percentage pain relief and obtained trial pain relief (rs = 0.18, *p* = 0.08) and percentage pain relief and number of previous surgeries (rs = 0.17, *p* = 0.096). The correlation matrix is presented in Figure 3.

Through univariate analysis, a statistically significant relation between responder status and predominant pain location was revealed (Type II test 6.84, *p* = 0.03). Based on feature selection, trial pain relief, predominant pain location, and the number of previous surgeries were used in machine learning techniques to predict responder status based on pain relief.

When working with the definition of responder status based on a decrease in medication use, Spearman rank correlations revealed three statistically significant correlations between percentage medication use reduction and baseline MQS score (rs = 0.29, *p* = 0.004), percentage medication use reduction and baseline ODI score (rs = 0.28, *p* = 0.025) and percentage medication use reduction and trial pain relief (rs = 0.26, *p* = 0.011). Through univariate analysis, a statistically significant relation between responder status and predominant pain location was revealed (Type II test 10.94, *p* = 0.004), between responder status and baseline MQS score (Type II test 6.38, *p* = 0.011), between responder status and trial pain relief (Type II test 4.59, *p* = 0.03), and between responder status and smoking status (Type II test 5.21, *p* = 0.02). Based on feature selection, trial pain relief, predominant pain location, smoking status, baseline MQS score, and baseline ODI score were used in machine learning techniques to predict responder status based on medication use.

### 3.3. Machine Learning

An overview of all results of the machine learning techniques can be found in Table 2.

When defining a responder as a person with at least 50% pain relief, the combined stepwise forward and backward logistic regression approach resulted in a final model with predominant pain location as only statistically significant main effect (χ^2^ = 6.59, *p* = 0.037). The expected odds of being a responder was 0.54 (95% CI from 0.31 to 0.93) for a patient with predominant back pain. The odds of being a responder were about 1.18 (95% CI from 0.39 to 3.51) times higher for patients with a combination of back and leg pain and 4.01 (95% CI from 1.32 to 12.16) times higher for patients with predominant leg pain than for patients with only a back pain component. Logistic regression and classification trees resulted in the lowest accuracy to predict the responder status of patients in the validation set. Boosting, random forest, and bagging outperformed the other models with an accuracy and area under the curve of 58.33%.

When defining a responder as a patient with at least 30% pain relief, logistic regression resulted in a model with the number of previously performed surgeries as only main predictor (χ^2^ = 3.19, *p* = 0.07). For a patient without previous surgeries, the expected odds of being a responder is 1.11 (95% CI from 0.52 to 2.38). The odds of being a responder increased with a factor 1.29 (95% CI from 0.95 to 1.76) for each additionally performed surgery. This model demonstrated an accuracy of 70.83% and area under the curve of 50% on the validation set. Exactly the same predictions were obtained with LDA and classification trees. Boosting, however, reached a higher value for the area under the curve compared to the latter models. Random forest and bagging performed slightly worse compared to the other models.

**Figure 4:**
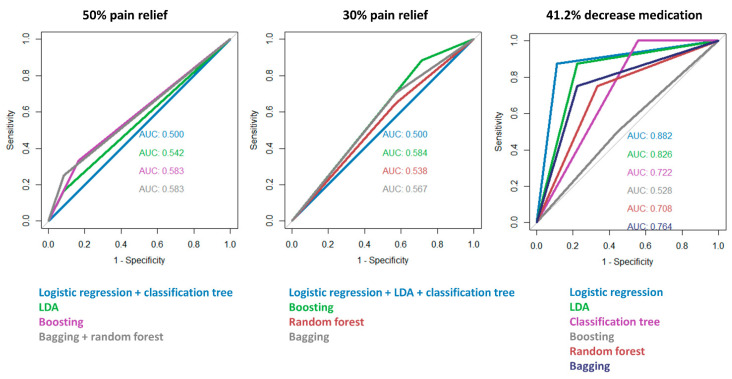
The receiver operating characteristics (ROC) curves of the different machine learning classification models for responders defined as 50% pain relief (left), 30% pain relief (middle) and 41.2% pain medication use reduction (right).

When defining a responder as a patient with at least 41.2% decreased in pain medication use, the stepwise procedure resulted in a logistic regression model with an MQS baseline score (χ^2^ = 5.84, *p* = 0.016), predominant pain location (χ^2^ = 8.88, *p* = 0.012), smoking status (χ^2^ = 6.49, *p* = 0.01), percentage trial pain relief (χ^2^ = 3.99, *p* = 0.046), and an interaction and main effect between the MQS baseline score and percentage trial pain relief (χ^2^ = 2.14, *p* = 0.14). For a one unit increase in MQS score, the odds of being a responder increased by a factor of 1.62 (95% CI from 0.98 to 3.12). For a one unit increase in percentage trial pain relief, the odds of being a responder increase by a factor of 1.09 (95% CI from 1.01 to 1.21). The increase in odds of being a responder per extra unit increased in pain medication use, and decreased with a factor 0.99 (95% CI: 0.986–1.002) for each 1 unit increase in percentage trial pain relief. The expected odds of being a responder when having back and leg pain and when having predominant leg pain increase with a factor 0.000 (95% CI from 0.000 to inf.) and 1.55 (95% CI from 0.39 to 6.91), respectively. The expected odds of being a responder decreased with a factor 0.18 (95% CI from 0.04 to 0.68) when being a smoker. This model had an accuracy of 88.24% and area under the curve of 88.19%. LDA and bagging were also performing well with an accuracy of 82.35% and 76.47% and area under the curve of 82.64% and 76.39% on the validation set, respectively. Figure 4 presents the ROC curves for all models.

## 4. Discussion

A broad gamma of mathematical/statistical models were created to predict responders to HF-10 SCS with information available before definitive SCS implantation in patients with FBSS. Several techniques resulted in models with an acceptable accuracy to predict HF-10 SCS responders with both 30% and 50% pain relief as cut-off values. However, none of the created models obtained an excellent accuracy on the validation set. This study thus demonstrated that it remains challenging to predict the responder status to HF-10 SCS with currently available pre-operative factors. This result seems to be in line with a previous study denoting that there were no predictive preoperative factors to determine the outcome of SCS in terms of pain relief [24].

One of the most surprising results of this study were the weak correlations between percentage pain relief as outcome variable and baseline clinical factors. None of the available clinical variables revealed a strong relationship with the outcome variable. Moderate correlations were revealed between total ODI score and medication intake. Previous research already revealed that achieving functional goals is more important to patient satisfaction than obtaining reduction in self-reported pain [25]. Rather recently, the focus has shifted towards functioning instead of only focusing on pain relief, with multidimensional outcome measures such as holistic responders [26] or composite scores [27]. Another important issue in light of the ongoing opioid epidemic is the reduction in medication use. Therefore, a third definition of responder was created based on reaching the minimal clinical important difference of the MQS [23]. With this outcome, logistic regression and LDA resulted in an accuracy of more than 80% on the validation set. These results indicate that it might be easier to predict whether a patient will take less pain medication at long-term follow-up than predicting whether their pain will be reduced.

In this study, feature selection was performed with correlation analyses and univariate analyses. In the literature, a broad range of different feature selection methods are available [28], whereby the exact method could induce an influence on the final results in terms of accuracy [29]. Feature selection clearly revealed the importance of trial pain relief in relation to percentage pain relief with HF-10 SCS at long-term. A wide variety in SCS trial periods were found between countries, ranging from the test stimulation of a few minutes immediately prior to permanent implantation up to SCS trial periods of 28 days [30,31]. Recently, a randomized controlled trial was conducted towards the clinical utility of a screening trial for SCS whereby the authors questioned the rationale behind a trial period due to limited evidence, limited clinical value, increased risk for the patient, and higher healthcare costs [32]. The results indicated that a SCS screening trial did not provide superior patient outcomes and was not cost-effective compared to no SCS trial [32]. In the current retrospective study, the percentage pain relief obtained during a SCS trial period of 28 days in a specific population, namely patients with FBSS, seemed to be related to the obtained pain relief at long-term follow-up, which is in contrast to the results of the randomized controlled trial. Nevertheless, this result should be interpreted carefully due to the low correlation coefficient. In Belgium, an SCS trial period is mandatory according to reimbursement rules; however, based on the results of the TRIAL-STIM study and the low correlation coefficients in the current study, a more in depth evaluation of the use of a trial period and its predictive value for success and reimbursement rules seems highly warranted.

The models in this study were created based on information that is collected in clinical routine care. The number of questionnaires that is routinely collected in daily care is often limited. More specifically, in this study, no questionnaires were included concerning psychological profiling. Previous studies, however, have been suggestive for a potential influence of psychological factors on the success of SCS [6,7]. Similarly, systematically collecting more precise information about the underlying pain mechanisms may improve prediction models. Additionally, for a lot of pre-operative evaluations, clinicians are relying on a single evaluation (i.e., “snapshot medicine”). To further improve the accuracy of prediction models, clinicians could gather pre-operative information on several time points to obtain more reliable self-reporting information. For example, pain intensity diaries over 7 days could be implemented in routine clinical care. Hypothetically, the lower accuracy for predicting responders for pain relief compared to predicting responders for decreased medication use could be explained by the subjective and perhaps highly influenced reporting of pain intensity, whereas medication use is based on more stable reporting. Moreover, it might be possible that self-reported variables are not specific enough to predict the outcome for long-term follow-ups. It has been previously suggested that self-reported and objective measurements are not in agreement with each other [33]. The currently measured variables were primarily self-reported variables, whereby it might be possible that more objective measurements are necessary to capture the full presentation of a patient and to enable a proper prediction of the outcome status. Disability was routinely measured with the ODI, while more objective measurement tools such as wearable activity trackers could provide complementary information that might be needed to enable an accurate prediction of the long-term response to HF-10 SCS [34]. Finally, data from baseline and the last available study visit was used. This was in contrast to prospective study designs wherein a predetermined study visit frame was designed for follow-up visits. In this retrospective study, the median time between the SCS trial and the last follow-up visit was 591.5 days, leading to a variable duration that SCS was applied as treatment. It may be possible that the lack of a narrow time frame limited the accuracy of the statistical models.

## 5. Conclusions

Several machine learning algorithms were applied to predict responders to HF-10 SCS with baseline clinical variables. None of the machine learning techniques reached a high accuracy to predict responders for pain relief on the validation set. Higher accuracies were obtained for predicting responders for pain medication decrease. The inconsistent results regarding predictive factors in the literature, combined with the acceptable accuracy of the currently obtained models, might suggest that routinely collected baseline parameters from clinical practice are not sufficient to consistently predict the SCS response at long-term with a high accuracy. It may be possible that systematically collecting objective outcome parameters could provide complementary information, which could result in models with better predictive performance.

## Figures and Tables

**Table 1:** Patient characteristics.

Variable	Measurement at Baseline	Measurement at Last Visit
Age (years)	51.5 (SD: 10.11)	
Sex (%)	Males: 41 (34.45%) Females: 78 (65.55%)	
Back pain intensity (*N* = 119)	No pain: 0 Mild pain: 2 (1.68%) Moderate pain: 12 (10.08%) Severe pain: 105 (88.24%)	No pain: 10 (8.40%) Mild pain: 27 (22.69%) Moderate pain: 45 (37.81%) Severe pain: 37 (31.10%)
Leg pain intensity (*N* = 118 for baseline)	No pain: 7 (5.93%) Mild pain: 14 (11.86%) Moderate pain: 22 18.64%) Severe pain: 75 (63.56%)	No pain: 33 (27.73%) Mild pain: 40 (33.61%) Moderate pain: 18 (15.13%) Severe pain: 28 (23.53%)
Pain location (*N* = 119)	Back: 75 (63.02%) Leg: 21 (17.65%) Back and leg: 23 (19.33%)	
EQ5D-3L (*N* = 69)	0.24 (Q1–Q3: 0.19–0.39)	0.58 (Q1–Q3: 0.29–0.66)
EQ5D VAS (/100) (*N* = 78)	30 (Q1–Q3: 20–50)	60 (Q1–Q3: 40–75)
MQS (*N* = 118)	7.3 (Q1–Q3: 3.4–14.62)	3.4 (Q1–Q3: 0–9.48)
ODI (/100) (*N* = 81)	58.6 (SD: 13.94)	42.25 (SD: 16.53)
BMI (*N* = 119)	27.08 (Q1–Q3: 23.66–30.37)	
HR (bpm) (*N* = 119)	75.49 (SD: 12.40)	
SBP (*N* = 119)	133 (Q1–Q3: 117–142.5)	
DBP (*N* = 119)	80 (Q1–Q3: 71–89)	
MAP (*N* = 119)	97 (Q1–Q3: 88.83–105.83)	
Surgeries (*N* = 119)	2 (Q1–Q3: 1–3)	
Pain onset (*N* = 119)	New: 16 (13.45%) Recurrent: 16 (13.45%) Remaining: 87 (73.11%)	
Pain duration (*N* = 119)	6.5 (Q1–Q3: 4–12) years	
Smoking (*N* = 119)	No: 66 (55.46%) Yes: 53 (44.54%)	
Work (*N* = 119)	Yes: 23 (19.33%) Financial compensation: 20 (86.96%) No financial compensation: 3 (13.04%) No: 96 (80.67%) Worker’s compensation: 77 (80.21%) No worker’s compensation: 19 (19.79%)	
Marital status (*N* = 119)	Married: 71 (59.66%) Single: 23 (19.33%) Living together: 19 (15.97%) Living apart together (LAT relation): 6 (5.04%)	
Children (*N* = 119)	Yes: 59 (49.58%) No: 60 (50.42%)	

Abbreviations. BMI: body mass index, DBP: diastolic blood pressure, EQ5D-3L: the EuroQol with five dimensions and three levels, HR: heart rate, MAP: mean arterial pressure, MQS: medication quantification scale, ODI: Oswestry Disability Index, SBP: systolic blood pressure, VAS: visual analogue scale.

**Table 2:** Comparison between machine learning techniques applied on the validation set.

	Accuracy	Area under the Curve	Prediction/Actual Status			
					0	1
			0		TN	FN
			1		FP	TP
**Responder 50% pain relief**						
Logistic regression	50.00%	50.00%	11	11		
			1	1		
LDA	54.17%	54.17%	11	10		
			1	2		
Classification tree	50.00%	50.00%	10	10		
			2	2		
Boosting	58.33%	58.33%	10	8		
			2	4		
Random Forest	58.33%	58.33%	11	9		
			1	3		
Bagging	58.33%	58.33%	11	9		
			1	3		
**Responder: 30% pain relief**						
Logistic regression	70.83%	50.00%	0	0		
			7	17		
LDA	70.83%	50.00%	0	0		
			7	17		
Classification tree	70.83%	50.00%	0	0		
			7	17		
Boosting	70.83%	58.40%	2	2		
			5	15		
Random Forest	58.33%	53.78%	3	6		
			4	11		
Bagging	62.50%	56.72%	3	5		
			4	12		
**Responder: 41.2% decrease medication use**						
Logistic regression	88.24%	88.19%	8	1		
			1	7		
LDA	82.35%	82.64%	7	1		
			2	7		
Classification tree	70.59%	72.22%	4	0		
			5	8		
Boosting	52.94%	52.78%	5	4		
			4	4		
Random Forest	70.59%	70.83%	6	2		
			3	6		
Bagging	76.47%	76.39%	7	2		
			2	6		

The confusion matrix presents a table with four different combinations of predicted and actual values to present the performance measurement for machine learning classification problems. A value of zero is coding a non-responder and 1 is recoding a responder in the confusion matrix. True positive and true negatives are observations that are correctly predicted by the model. False positives are observations for which the model predicted a responder whereas in reality it is a non-responder. False negatives are observations for which the model predicted a non-responder whereas in reality it are responders. Based on the confusion matrix, accuracy is calculated with the following formula: ((true positives + true negatives)/total number of positives and negatives). Note that the validation set for responders based on medication use is smaller (*n* = 17) due to less observations for baseline ODI score. Abbreviations. FN: false negatives, FP: false positives, LDA: Linear Discriminant Analysis, TN: true negatives, TP: true positives.
